# Effect of Hot Water Treatment Combined With Carrageenan Coating on Physicochemical Parameters and Disease Resistance of Banana Fruit

**DOI:** 10.1002/fsn3.70333

**Published:** 2025-06-02

**Authors:** Gemechu Warkina Lencho, Nguyen Thi Hanh

**Affiliations:** ^1^ Department of Food Science and Postharvest Technology Jimma University College of Agriculture and Veterinary Medicine Jimma Ethiopia; ^2^ Department of Postharvest Technology Faculty of Food Science and Technology, Vietnam National University of Agriculture Vietnam

**Keywords:** banana, carrageenan, disease resistance, hot water treatment, ripening, shelf life

## Abstract

Banana is an important climacteric fruit because of its nutritional values and bioactive components. However, it is a perishable fruit which has a short shelf life; so, an improved postharvest method to delay its ripening is required. The impact of dipping bananas in hot water at 47°C for 5 min, combined with a 0.5% carrageenan coating, during storage at 13°C and 85%–90% relative humidity was studied using a completely randomized design (CRD) with three replications. The results showed that the combined treatment significantly maintained physicochemical parameters and suppressed the fruit disease incidence as compared with individual treatment and untreated fruits. Also, the highest total phenolic content and radical scavenging activity were observed in the combined treatment of ripe banana peel (0.813 g GAE/kg FW and 59.05%, respectively) on the 21st day of storage. However, the lowest values were observed in the untreated fruits. The findings showed that the stage at which bananas ripen affected their phenolic content and antioxidant activity. The combination of hot water treatment and carrageenan coating maintained lower weight loss and greater peel color hue values. The combined treatment successfully postpones fruit ripening by maintaining fruit firmness, reducing the steady rise in total soluble solids (TSS), and delaying the decline in the titratable acidity of fruit pulp. The overall findings indicated that the application of combined treatment could be used to lower the disease development and preserve the fresh quality through an effective strategy to suppress postharvest decay and delay the ripening of banana fruits.

## Graphical abstract

Generally, the findings of this study indicated that the application of combined treatment could be used to lower disease development and preserve fresh quality through an effective strategy to suppress postharvest decay and delay the ripening of banana fruits.

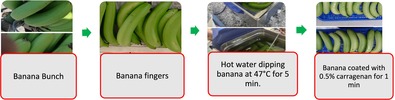

## 1 Introduction

Banana (*Musa* spp.) is one of the most extensively cultivated and consumed fruit crops in the world. After rice, wheat, and maize, bananas are the world's fourth‐largest food crop (Voora et al. 2020). Bananas have a large amount of resistant starch, which functions as dietary fiber in the body. They can also be a significant source of naturally occurring antioxidants, such as ascorbic acid and polyphenol compounds (Aquino et al. 2016). However, bananas are climacteric fruits that ripen at the postharvest stage because of the rise in respiration and ethylene production. Therefore, the fruit deteriorates quickly throughout the postharvest period.

Several other technologies have been implemented with varying degrees of success to preserve the quality and extend the shelf life of fruits and vegetables, either separately or in combination. Edible coatings and hot water treatment are safe ways to preserve fruits and vegetables and increase their shelf life (Fallik 2004; Lin and Zhao 2007). Research has examined the application of edible coating in conjunction with hot water treatment to maintain fruit quality and minimize unanticipated damage (Ban et al. 2015). A coating substance called carrageenan has been used on fruits and vegetables to lessen oxidation of chemicals, water loss, aging, and the growth of bacteria. Carrageenan has been used as an alternative method of edible coating to delay ripening and extend the shelf life of banana fruits. It affected gene expressions probably due to the limited O_2_ concentration in the cell as the consequence of the presence of a gas barrier (Dwivany et al. 2020).

However, Dissanayake et al. (2015) have observed that hot water treatment at 35°C and 40°C for 5 min inhibits the ripening and loss of green color of Seeni Kesel bananas during ambient storage. Nevertheless, no published information exists about the use of carrageenan coating in combination with hot water treatment to preserve fruit quality and extend banana shelf life. Therefore, the present study evaluated the impact of hot water treatment combined with carrageenan coating on the physicochemical parameters and disease resistance of banana fruit during ripening.

## 2 Materials and Methods

### 2.1 Experimental Material Preparation

*Musa acuminata*, AAA group, mature green bananas were collected from the farm in Le Chi commune, Gia Lam district, Hanoi, Vietnam. The bunches were transported to the laboratory within one hour after harvesting. Upon the arrival to the laboratory, fruits at stage I, according to Thompson et al. (2019), color scale without blemishes or physical damage were selected, cut into individuals, and cleaned with tap water for the experiment.

The disaccharide repeat unit of k‐carrageenan (sulfated plant polysaccharide, Sigma Chemical Co., USA) is made up of residues of (1,3)‐β‐galactopyranose‐4‐sulfate and (1,4)‐α‐3,6‐anhydrogalactopyranose. It weighs 788.647 g/mol at the molecular level.

### 2.2 Chemicals

Gallic acid (Wako Pure Chemical Industries Ltd., Osaka, Japan), polyethylene glycol 200, and glycerol (Promega Co., USA), Folin ciocalteau (Sigma Aldrich, India), sodium carbonate, phenolphthalein indicator, methanol (Analytical grade, Belgium), DPPH (Sigma Chemical Co., USA), acetone (Xilong scientific Co. Ltd., China), NaOH (Analytical grade, China), ethanol (Analytical grade, China). Every other reagent was of analytical quality. Distilled water was used to create standard solutions.

### 2.3 Making the Solution for the Carrageenan Coating

For the preparation of the coating, 0.5 g of carrageenan powder was dissolved in 100 mL of distilled water, according to Lee et al. (2003). A plasticizer was made by mixing PEG 200 and glycerol 50:50 (w/w) (0.75 g/g weight of carrageenan powder). On a hot plate, the solutions were vigorously agitated for 40 min using a magnetic stirrer bar after being equilibrated at 70°C. After cooling to ambient temperature, the solutions were kept at 20°C before using them. Carrageenan coating was carried out by dipping the fruits in a 0.5% carrageenan solution for 1 min.

### 2.4 Design of Experiment and Use of Treatments

**FIGURE 1:**
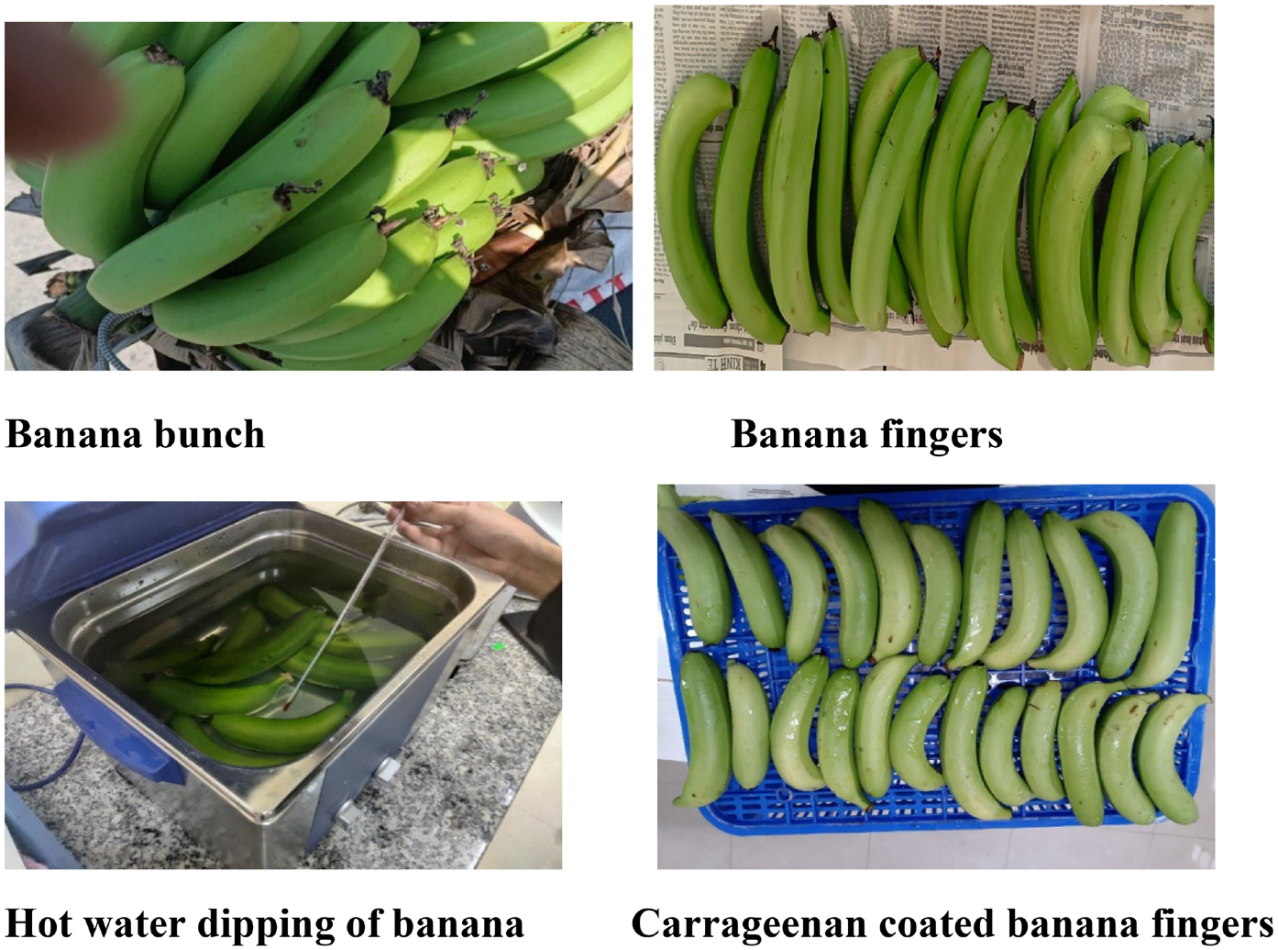
Sample preparation.

A completely randomized design (CRD) with three replications was used to carry out the experiment. Preliminary tests were used to select the suitable carrageenan concentration and hot water treatment. There were four treatments: control, hot water treatment at 47°C for 5 min, 0.5% carrageenan coating, and hot water treatment + carrageenan coating (combined treatment). Fruits were initially dipped in hot water at 47°C for 5 min as part of the combined treatment. Banana fruits were then air‐dried at room temperature and stored at 13°C and 85%–90% relative humidity after being coated with 0.5% carrageenan (Figure 1). In total, 360 fruits were used for the experiment. The parameters measured included peel color, browning score, physiological weight loss, disease incidence, disease severity, shelf life, firmness, TSS, TA, total chlorophyll content of peel, phenolic content, and antioxidant activity. Data were collected for the analysis within 7 days of the interval during storage.

### 2.5 Analytical Method

#### 2.5.1 Determination of Changes in Banana Peel Color

A colorimeter (model CR‐400, Minolta, Japan) was used to measure the hue angle value in order to determine variations in peel color. Three parts of each fruit were measured (Varit and Songsin 2011). Each replication utilized five fruits.

$$H^{o}=180^{0}+\tan^{-1\left(b*/a*\right)}\;\text{if}\;a^{*}<0$$

$$\tan^{-1\left(b*/a*\right)}\;\text{if}\;a^{*}<0$$

The hue values of 0°, 90°, 180°, and 270° correspond to the red, yellow, green, and blue colors, respectively.

#### 2.5.2 Peel Browning Measurement

The following scale was used to measure the overall brown area of each fruit surface in order to assess the fruit's browning: According to the Ding and Ling (2014) technique, 1 = indicates no browning, 2 = indicates less than 20% of the banana peel surface, 3 = indicates 20%–40% of the banana peel surface, 4 = indicates 40%–60% of the banana peel surface, and 5 = indicates more than 60% of the banana peel surface.

#### 2.5.3 Physiological Weight Loss

Weight loss was determined by using a portable electronic scale balance. The following formula was used to compute the percentage of physiological weight loss according to Abiso et al. (2015) method. Each replication utilized five fruits.

$$\mathrm{PWL}\;\left(\%\right)=\frac{\left[\text{Initial weight}\;\left(g\right)–\text{final weight}\;\left(g\right)\right]}{\text{Initial weight}\;\left(g\right)}\times 100$$

#### 2.5.4 Pulp Firmness

Varit and Songsin (2011) used a penetrometer to test the firmness of the fruit in Newtons (N). Before applying a probe (5 × 10–3 m) to the fruit (three portion per fruit), the fruit's peel was removed.

#### 2.5.5 Total Soluble Solid Content

Total soluble solid (°Brix) was determined by AOAC method using a digital refractometer (PAL 1, Atago Co Ltd) by placing a drop of juice on its prism (AOAC 2012). Three fruits were used in each replication.

#### 2.5.6 Banana Pulp Titratable Acidity

Titratable acidity (%) was determined by the method AOAC (2012). Titratable acidity was expressed as a percentage of fresh weight.

#### 2.5.7 Total Chlorophyll Content

The Arnon (1949) method was used to measure the amount of chlorophyll. Twenty milliliter of 80% acetone were used to homogenize a 5‐g sample of banana peel tissue. After passing the mixture through filter paper and adjusting the volume to 100 mL with 80% acetone, spectrophotometric measurements were made at 663 and 645 nm. Thereafter the total chlorophyll content was determined and reported as mg/g FW.

#### 2.5.8 Total Phenolic Content

Total phenolic content in banana pulp and peel was measured using the Folin‐Ciocalteau method (Singleton et al. 1999). Three grams of fresh samples was extracted with 25 mL of 80% methanol; then homogenized and filtered with filter paper. 0.15 mL of the sample extract, 2.5 mL of distilled water, and 0.15 mL of fresh Folin‐Ciocalteau solution were added. The sample was incubated for 3 min before 300 μL of 1 N sodium carbonate solution was added. Samples were then incubated for 2 h at 23°C in a dark environment.

The similar protocol was used to create a standard curve using a series of gallic acids. Gallic acid standard solution and the calibration curve were generated from various concentrations (0, 20, 40, 60, 80, and 100 μg/mL) of gallic acid. All reagents except the standard and sample are present in the blank solution. A Shimadzu, Japan‐made UV 1601 spectrophotometer was used to measure the absorbance at 725 nm. Next, g of gallic acid equivalent (GAE) per kilogram of fresh weight was used to represent the total phenolic content.

#### 2.5.9 Antioxidant Activity

The DPPH technique was used to assess the antioxidant activity of banana peel and pulp (Li et al. 2017). Using a homogenizer, 3 g of fresh samples were extracted with 25 mL of 80% methanol. Using filter paper, the homogenate solution was filtered. Next, 150 μL of supernatant was added to 2850 μL of fresh working solution containing 0.12 mM DPPH, and the mixture was incubated for 30 min under dark conditions. A Shimadzu UV‐1601 and Japan UV–visible spectrophotometer was used to measure the solution's absorbance at 517 nm. The following formula was used to determine the DPPH scavenging activity:

$$\%\text{DPPH scavenging}=\frac{\left(A0-A1\right)}{A0}\times 100$$

where, A_0_ is the control absorbance; A_1_ is the sample absorbance.

#### 2.5.10 Disease Incidence and Severity

The number of infected fruits and the total quantity of fruits in each treatment were used to calculate the disease incidence. Eye estimate can be used to visually identify the sick fruits (Monerzumma et al. 2009).

$$\text{Disease incidence}\;\left(\%\right)=\frac{\text{Number of banana infected fruits}}{\text{Total number of banana fruits}}\times 100$$

Eye estimate was used to determine the disease severity, which is the percentage of the infected fruit that is diseased. The following scale was used to rate the severity of the disease in accordance with Rinaudo's (2006) methodology: 1 = represents 0% of the fruit's surface rottenness, 2 = represents 1%–25%, 3 = represents 26%–50%, 4 = represents 51%–75%, and 5 = represents 76%–100% of the fruit's surface rottenness.

#### 2.5.11 Shelf Life

The number of days needed from harvest to unfit for consumption was used to determine the shelf life of banana fruits as affected by all treatments and untreated bananas (Mondal 2000).

### 2.6 Statistical Data Analysis

The data were assessed for the impact of the treatments on the parameters under investigation using the analysis of variance (ANOVA) method. According to Snedecor and Cochran (1980), the treatment averages were separated and compared using the least significant differences (LSD) at the 0.05 level of significance. All statistical analyses were conducted using SPSS 16.0 software (SPSS Inc., Chicago, IL, USA). The means ± standard deviation (STDEV) was used to display the data.

## 3 Result and Discussion

### 3.1 Effect of Hot Water Combined With Carrageenan Coating on Physicochemical Parameters

The impact of hot water treatment and carrageenan coating on the physicochemical characteristics of banana fruit during cold storage is displayed in Table 1. In comparison with untreated banana fruits or each treatment alone, the results showed that the combination of hot water treatment and carrageenan coating maintained a greater value of flesh hue angle (h°), fruit pulp hardness, and decreased weight loss. Additionally, by keeping the fruit firm, reducing the steady rise in total soluble solids (TSS), and delaying the decline in titratable acidity (TA), the combination treatment and carrageenan coating alone postponed fruit ripening. Khalil et al. (2022) discovered that mango ( *Mangifera indica* L. cv. Kent) fruits found comparable results when hot water treatment was combined with chitosan coating.

**TABLE 1:** Effect of hot water treatment combined with carrageenan coating on physicochemical parameters of banana fruit.

Storage time (days)	Treatment	Weight loss (%)	Peel color (Hue value)	Firmness (N)	TSS (^o^Brix)	TA (%)
0	CONTROL	0.00	115.15 ± 0.422^a^	29.58 ± 0.539^a^	5.07 ± 0.404^a^	0.60 ± 0.030^a^
	HWD	0.00	114.9 ± 0.015^a^	29.58 ± 0.539^a^	5.07 ± 0.404^a^	0.60 ± 0.030^a^
	0.5% CAR.	0.00	114.87 ± 0.064^a^	29.58 ± 0.539^a^	5.07 ± 0.404^a^	0.60 ± 0.030^a^
	HWD + CAR.	0.00	114.87 ± 0.064^a^	29.58 ± 0.539^a^	5.07 ± 0.404^a^	0.60 ± 0.030^a^
	Significance	0.00	NS	NS	NS	NS
	LSD (0.05)	0.00	1.38	4.54	0.76	0.06
	CV %	0.00	0.12	1.82	7.97	5.00
7	CONTROL	4.11 ± 0.109^a^	110.98 ± 0.261^c^	20.36 ± 1.539^b^	9.43 ± 0.289^a^	0.41 ± 0.052^ab^
	HWD	2.36 ± 0.363^c^	111.57 ± 0.183^c^	23.73 ± 2.111^ab^	6.87 ± 0.404^bc^	0.51 ± 0.066^a^
	0.5% CAR.	2.90 ± 0.367^b^	112.24 ± 0.226^b^	25.61 ± 2.634^a^	6.23 ± 0.874^c^	0.39 ± 0.064^b^
	HWD + CAR.	2.58 ± 0.213^bc^	113.32 ± 0.497^a^	27.32 ± 1.370^a^	7.43 ± 0.252^b^	0.50 ± 0.027^a^
	Significance	*	*	*	*	*
	LSD (0.05)	0.54	0.59	3.72	0.98	0.10
	CV %	8.80	0.26	8.31	6.07	11.54
14	CONTROL	7.03 ± 0.612^a^	92.04 ± 0.594^d^	9.37 ± 0.499^c^	14.87 ± 0.115^a^	0.26 ± 0.008^b^
	HWD	6.07 ± 0.463^b^	94.31 ± 1.050^c^	11.54 ± 1.799^bc^	14.57 ± 0.53^ab^	0.30 ± 0.020^a^
	0.5% CAR.	5.81 ± 0.464^b^	102.01 ± 0.444^b^	12.84 ± 0.352^b^	14.37 ± 0.208^ab^	0.27 ± 0.013^b^
	HWD + CAR.	4.65 ± 0.193^c^	104.71 ± 1.116^a^	16.32 ± 1.784^a^	14.10 ± 0.458^b^	0.33 ± 0.020^a^
	Significance	*	*	*	*	*
	LSD (0.05)	0.86	1.60	2.46	0.51	0.03
	CV %	7.00	1.00	8.85	1.61	5.26
21	CONTROL	10.01 ± 0.193^a^	84.61 ± 0.236^d^	8.39 ± 1.115^b^	17.60 ± 0.721^a^	0.23 ± 0.010^b^
	HWD	8.63 ± 0.135^b^	87.92 ± 0.329^c^	8.61 ± 0.468^ab^	14.73 ± 0.416^b^	0.25 ± 0.015^ab^
	0.5% CAR.	8.91 ± 0.141^b^	88.72 ± 0.397^b^	9.10 ± 0.337^ab^	14.57 ± 0.058^b^	0.25 ± 0.010^ab^
	HWD + CAR.	7.31 ± 0.337^c^	92.67 ± 0.388^a^	9.70 ± 0.381^a^	13.27 ± 0.416^c^	0.27 ± 0.015^a^
	Significance	*	*	*	*	*
	LSD (0.05)	0.41	0.65	1.24	0.88	0.02
	CV %	2.31	0.38	6.43	2.68	5.00
28	CONTROL	14.43 ± 0.164^a^	83.63 ± 0.542^d^	5.39 ± 0.738^b^	20.03 ± 1.331^a^	0.17 ± 0.014^d^
	HWD	11.70 ± 0.181^b^	85.20 ± 0.286^c^	6.32 ± 0.191^b^	21.10 ± 0.954^a^	0.21 ± 0.010^c^
	0.5% CAR.	11.67 ± 0.479^b^	86.20 ± 0.350^b^	7.37 ± 0.310^a^	19.37 ± 0.929^a^	0.23 ± 0.004^b^
	HWD + CAR.	10.01 ± 0.732^c^	87.54 ± 0.249^a^	7.87 ± 0.702^a^	18.87 ± 1.795^a^	0.25 ± 0.004^a^
	Significance	*	*	*	Ns	*
	LSD (0.05)	0.86	0.70	1.02	2.43	0.02
	CV %	3.23	0.42	7.20	6.31	3.72

*Note:* Means followed with the same letter(s) are not significantly different at *p* < 0.05; each column of the same storage day was analyzed separately.

Abbreviations: *, significant; CV, variation coefficient; LSD, least significant difference; NS, non‐significant.

When the storage period was progressed to 28 days, the percentage of weight loss in the treated fruits raised up gradually (Table 1). When compared to untreated fruits, all treatments significantly reduced the weight loss of banana fruits during cold storage (*p* < 0.05). The untreated fruits had the greatest weight loss (14.43%) at the end of storage, whereas the combined treatment had the lowest value (10.01%). On the final day of storage, fruits coated with carrageenan and mixed with hot water had the highest flesh hue angle values (87.54), followed by those coated with carrageenan and hot water (86.20 and 85.20, respectively). In contrast, the control treatment had the lowest flesh hue value (83.62), which was lower than the other treatments. There was a significant difference (*p* < 0.05) in hue value during storage due to treatments.

Data in Table 1 demonstrate how fruit hardness decreased over the course of the storage time for every treatment. Nevertheless, the control and hot water‐treated apples softened more quickly than the other treatments after 7 days of storage. Fruit firmness during storage varied significantly (*p* < 0.05) as a result of treatments. While the carrageenan‐coated and combined treated fruits displayed high fruit firmness values of 7.37 and 7.86 N, respectively, the control fruit and the fruit treated with hot water displayed lower values of 5.38 and 6.32 N, respectively, at the end of storage.

A climacteric fruit feature throughout the ripening process, titratable acidity (TA) decreased over the fruit's storage duration, whereas total soluble solids increased with time in all treatments (Table 1). Treatments resulted in a substantial change (*p* < 0.05) in both titratable acidity and total soluble solids during storage. All treatments considerably reduce the rise in TSS and fall in TA at the conclusion of the storage period when compared to the control. Additionally, on the final day of storage, fruits treated with carrageenan coating and combined treatment had the greatest TA values (0.23 and 0.25, respectively) and the lowest TSS values (19.36 and 18.86, respectively).

According to Salvador et al. (2007), the soluble solids content of the Cavendish cultivar grew in a quadratic fashion as it ripened. They discovered that soluble solids ranged from around 5.5% Brix (green) to 18% Brix (ripe) fruits, which verifies the conclusions of this experiment. Li et al. (2011) found a favorable correlation between the increase in the total sugar content of “Baxi” banana (*Musa* spp. AAA Group) fruit and the rise in sucrose phosphate synthase, sucrose synthase, and invertase activities during ripening. Similar to this, the TA content steadily dropped as the banana fruit known as “Gross Michel” (*Musa acuminate*, AAA group) ripened (Thaiphanit and Anprung 2010). On the other hand, Youryon and Supapvanich (2017) found that the mature green stage of *Musa* sp. AA group “Leb Mue Nang” banana fruit had a much lower TA level than ripe fruit, which was linked to a drop in pH.

### 3.2 Effect of Carrageenan Coating and Hot Water Treatment on Disease Resistance and Browning

**FIGURE 2:**
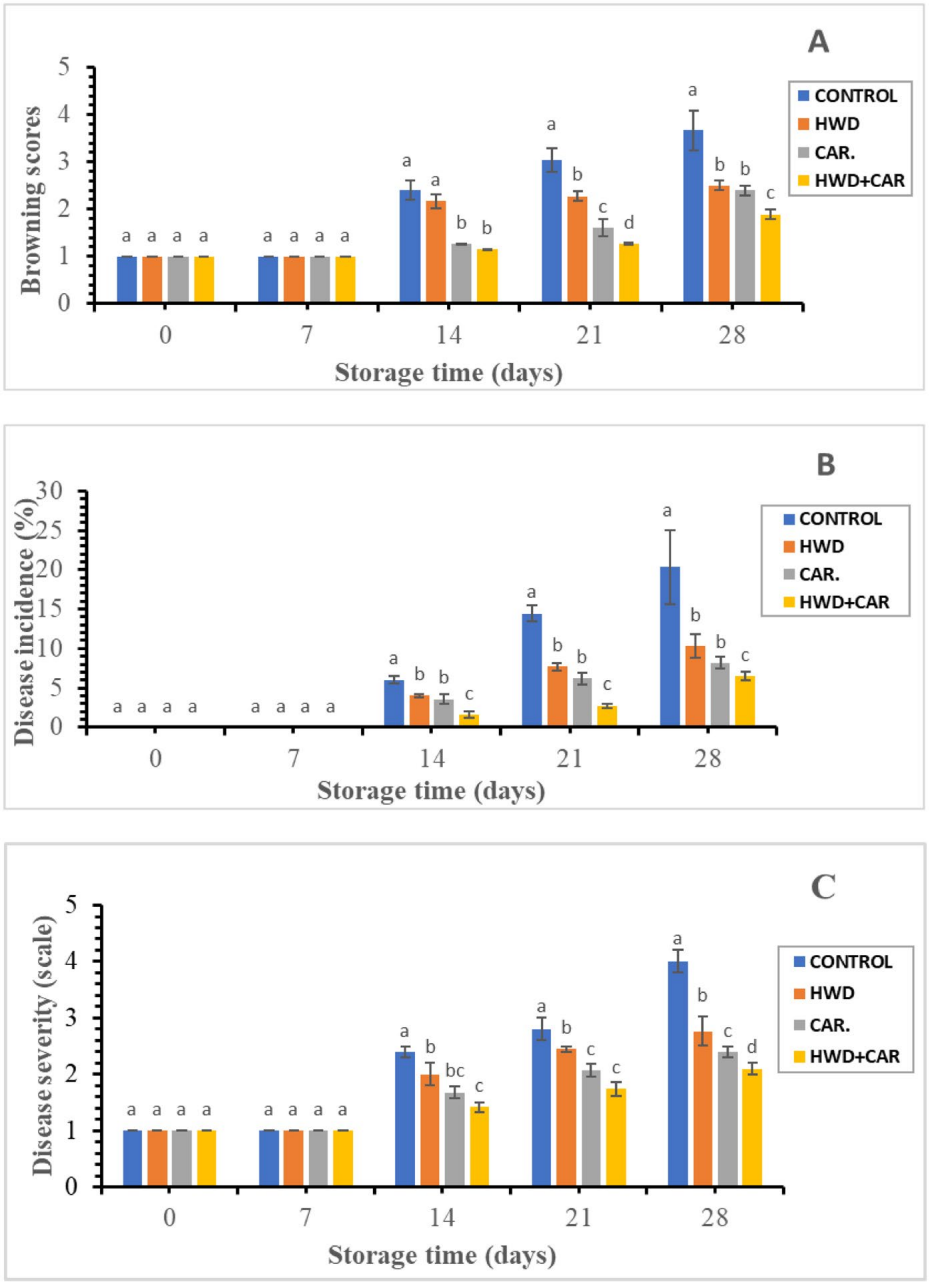
Effect of hot water combined with carrageenan coating on browning score (A), disease incidence (B), and disease severity (C) of banana fruit. Different letters above bars indicate significant differences at *p* < 0.05 on the same storage day according to LSD method.

According to Ding and Ling (2014), oxidative browning reactions, in which polyphenol oxidase converts phenolic chemicals into melanin, a brown pigment, are the cause of the brownish red coloring on the peel surface of berangan banana fruit. Figure 2A shows that either carrageenan or hot water treatment alone and combined treatment significantly inhibited browning of banana fruit compared to control. The surface of the banana peel did not discolor over the seven days of storage in any of the treatments. Following 14 days of storage, all treatments' browning scores increased as storage time progressed. The browning score of banana fruits during storage varied significantly (*p* < 0.05) depending on the treatments. There was a significant difference (*p* < 0.05) in browning score of banana fruits during storage due to treatments. At the end of storage time, the maximum average browning score was 3.67 (meaning, 20%–40% browning of banana peel surface) recorded in control, followed by hot water treatment 2.5, carrageenan coating score 2.4 (meaning, less than 20% browning of peel surface) according to this rating scale. However, the minimum average browning score 1.89 (no browning of peel surface) was observed in combined treatment according to this rating scale.

Figure 2B showed that all treatments reduced the rising disease incidence of banana fruits when compared to the control. Compared to the other treatments, the control banana fruits had a higher incidence of disease. The control treatment had the highest disease incidence at the end of the storage period (20.33%), followed by the fruits treated with hot water (10.33%). The carrageenan and combined treatments had the lowest disease incidences, at 8.16% and 6.5%, respectively. The outcome showed that, in comparison with the single treatment and the control, the combined treatment significantly (*p* < 0.05) reduced the fruit decay percentage. In support of this finding, Khalil et al. (2022) discovered that the control mango fruits had a higher percentage of deterioration than those treated with hot water and coated with chitosan after being stored for 28 days at 13°C ± 0.5°C and 85%–90% relative humidity (RH). Additionally, Kaka et al. (2019) found that the Basari banana fruit treated with hot water at 60°C for 10 min had the highest decay incidence (15.81%), followed by the control (15.38%).

Over the course of the 28‐day storage period, the banana fruits' disease severity progressively rose from Day 14 to the conclusion of storage (Figure 2C). The degree of illness in banana fruits during storage varied significantly (*p* < 0.05) depending on the treatments. On the last day of storage, comparatively, the highest score of disease severity was observed in control (untreated bananas) which was 4 (meaning 51%–75% of fruit surface was rotten) scored whereas the lowest score was recorded in combined treatment, which was 2.1 (only 1%–25% of rotten fruits) according to this rating scale. This finding suggests that the use of hot water treatment in conjunction with carrageenan coating may be responsible for the relatively decreased disease severity of bananas. This result is consistent with what Sikder and Islam (2019) found. According to their findings, banana fruit coated with 1% chitosan had the lowest disease severity score (1.8, meaning 0% of the fruit surface was rotten), while control samples had the highest disease severity score (4.76, meaning 51%–75% of the fruit surface was rotten).

### 3.3 Effect of Hot Water Combined With Carrageenan Coating on Chlorophyll Content

**FIGURE 3:**
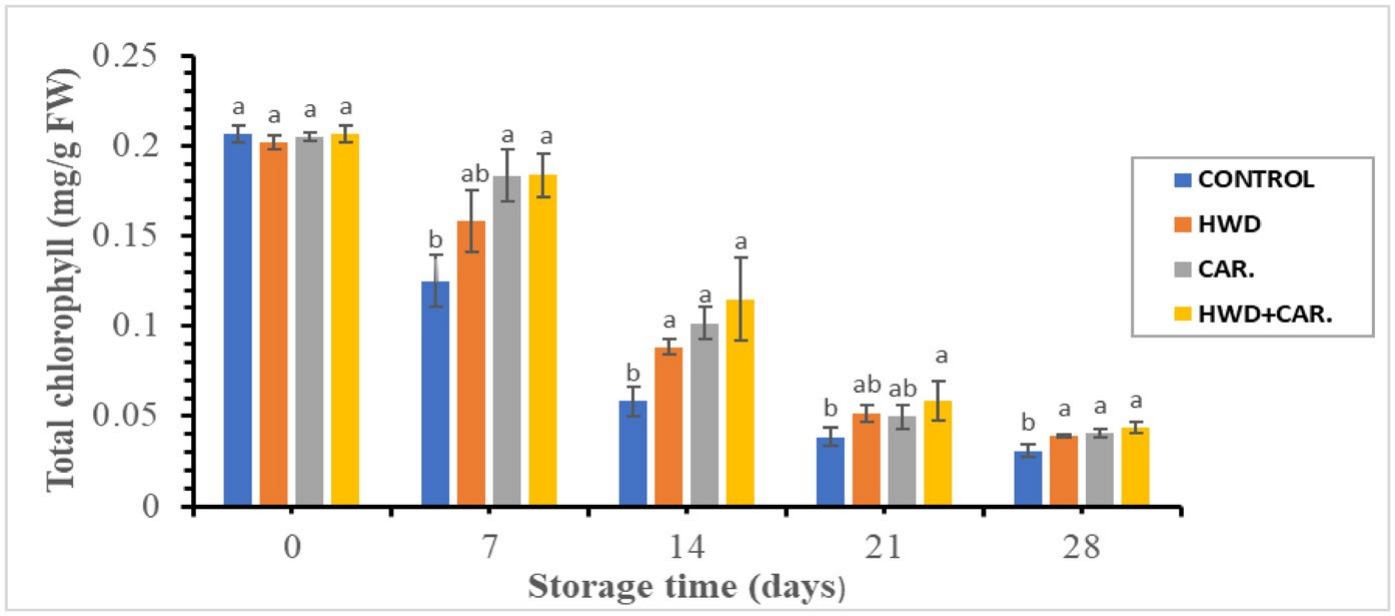
Effect of hot water combined with carrageenan coating on total chlorophyll content of banana peel. Different letters on the bars indicate significant differences at *p* < 0.05 on the same storage day according to the LSD method.

Figure 3 shows that the content of chlorophyll decreased rapidly in control fruits after 7 days of storage. However, it decreased slightly in treated banana fruits compared with control. There was a significant difference (*p* < 0.05) in total chlorophyll content of banana fruit during storage due to treatments. The maximum total chlorophyll content of banana (0.206 mg/g FW) was recorded at 0 days of storage and the lowest value (0.031 mg/g FW) was recorded at the last day of storage in untreated fruit. The result indicated that total chlorophyll degradation was inhibited in combined treatment compared to control and hot water or carrageenan coating alone. In agreement with this study, El‐Boray et al. (2015) found that the total chlorophyll content in William's banana peel between 0.414 and 0.022 mg/g FW. Additionally, Ding et al. (2007) found that the chlorophyll contents of Cavendish bananas (*Musa acuminata* 'Williams*'*) at 18°C ranged from 14.74 to 36.48 μg/g during ripening, which is lower than the values found in this study. According to Varit and Songsin (2011), dipping fruit in water at 50°C for 10 min decreased both the respiration rate and the rate at which chlorophyll degraded. According to Hailu et al. (2013), the most significant eating criterion that consumers use to assess when fruit is ripening is the peel's color changing from green to yellow due to chlorophyll breakdown.

### 3.4 Variations in Antioxidant Activity and Total Polyphenols

**FIGURE 4:**
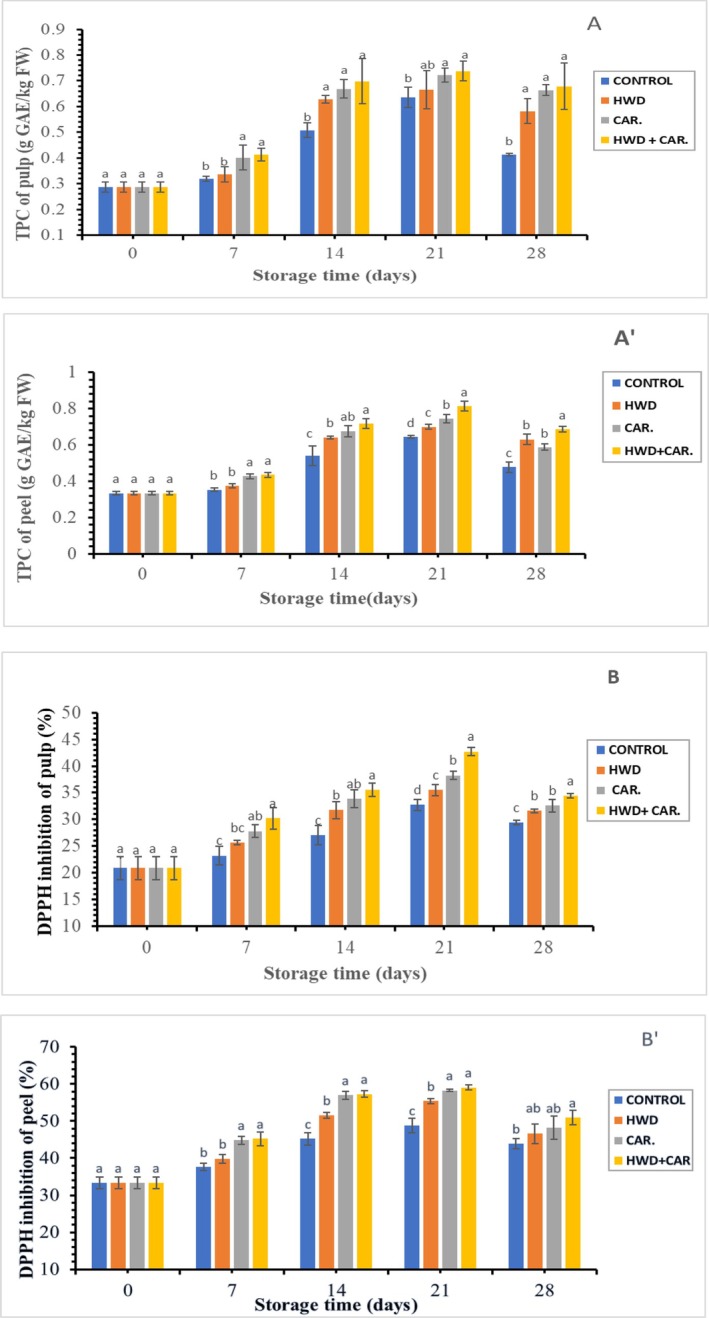
Effect of hot water combined with carrageenan coating on total polyphenol content (A, A’) and antioxidant activity (B, B’) of banana fruit. Different letters above bars indicate significant differences at *p* < 0.05 on the same storage day according to the LSD method.

Figure 4A,A’ illustrates that the total polyphenolic content (TPC) in banana fruits across all treatments tended to increase after being stored in cold storage. Besides, the phenolic content in peels was higher than that of banana pulp. The total polyphenol content of banana fruit during storage varied significantly (*p* < 0.05) as a result of the treatments. On day 21 of storage, the combined treatment showed the highest phenolic content (0.813 g GAE/kg FW) in ripe peel and 0.738 g GAE/kg FW in pulp, respectively. According to Aquino et al. (2016), the ripe peel of bananas had the highest concentrations of phenolic compounds, with mean contents that were 3.19, 2.15, and 1.57 times higher than those of the unripe pulp, unripe peel, and ripe pulp, respectively. They discovered that the phenolic content in the green and ripe pulp of the “Ouro” cultivar (AA) ranged from 27.98 to 73.28 mg GAE/100 g, respectively. But according to their findings, the high phenolic content in green and mature peels ranged from 29.02 to 78.98 mg GAE/100 g, respectively.

For ripe banana pulp (*Musa paradisiaca*), Alothman et al. (2009) found phenolic average values of 27.0–72.2 mg of GAE/100 g, which are comparable to the results of this investigation. During storage, phenolic compounds accumulated more in the fruit treated with hot water and carrageenan coating, or in combination, than in the control fruit. The subsequent ripening processes of bananas during storage may be impacted by the increase in free phenolics and flavonoids at the start of fruit development through hot water treatment.

According to Mura and Tanimura (2003), the activity of phenylalanine ammonium lyase, the enzyme that supports phenol synthase, is influenced by the rise in total polyphenol content, and it is more active during cutting and peeling. Peng et al. (2016) also found that heat treatment increased the phenolic content of strawberries via increasing the activity of phenylalanine ammonia lyase (PAL). Because of increased polymerization and tannin inactivation during ripening, cultivated bananas lose their strong flavor. Comparably, in the current investigation, the total polyphenol levels rose as the banana ripened to full ripeness before falling in overripe fruit.

Figure 4B,B’ shows the antioxidant activity increased in all treatments with ripening and then decreased in overripe fruit. There was a significant difference (*p* < 0.05) in antioxidant activity of banana fruit during storage due to treatments. Maximum percent of DPPH inhibition (42.72%, 59.05%) was recorded on the 21st day of ripening in combined treatment in ripe pulp and peel, respectively. In agreement with this result, methanolic extracts of banana peels had stronger DPPH antioxidant activity (30.82%–51.66%) than ethanolic extracts (25.44%–30.27%), according to Okolie et al. (2016). According to Fatemeh et al. (2012), the average radical elimination capability of Malaysian Cavendish and dream cultivars ranged from 26% to 52% for ripe pulp and unripe peel, respectively. Additionally, they noted that the DPPH scavenging percentages for Mas bananas ranged from 24.4% to 72.2%.

According to Fernando et al. (2014), variations in antioxidant component concentrations are typically the cause of antioxidant activity, which is expressed as radical scavenging activity, which varies with ripening stage. When fruit was overripe or senescent, DPPH activity dropped. Increased peroxidase activity has been associated with a greater capacity to scavenge free radicals, a decrease in lipid peroxidation, and a reduction in cellular membrane breakdown. These factors may have played a role in the ripening‐delaying impact of heat shock or brief heat treatment (Fallik 2004). According to Lo'Ay and El‐Khateeb (2018), banana fruit that was kept at low temperatures also showed higher quantities of antioxidants, such as glutathione and ascorbic acid. The combination of heat treatment and chitosan coating preserved the wolfberry's greater levels of ascorbic acid, total phenolic contents, and antioxidant potential, according to Ban et al. (2015).

### 3.5 Effect of Hot Water Combination With Carrageenan Coating on Storage Life

Perishable fruit shelf life is determined by combining aspects of food safety and quality, such as physical, chemical, and microbiological deterioration. The direct technique, which counts the days from harvest to the last edible point, was employed in this study to determine how long it takes for a product to decay and become unfit for consumption. The time frame that begins when fruit is harvested and continues until it begins to rot is known as the shelf life (Mondal 2000). It is the most crucial factor in the loss of the fruit's biological reaction and its fundamental quality.

**FIGURE 5:**
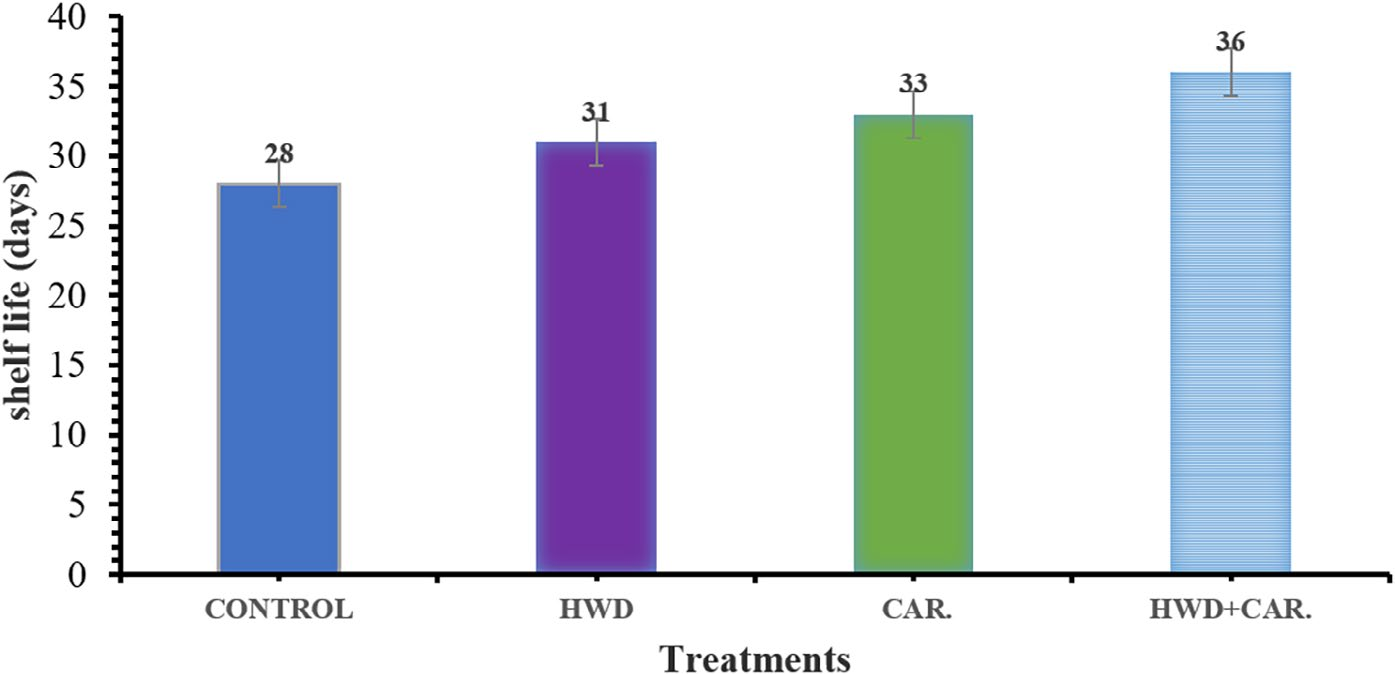
Effect of hot water combined with carrageenan coating on shelf‐life.

The impact of hot water treatment and carrageenan coating on the shelf life of banana fruit during cold storage at 13°C and 85%–90% relative humidity is shown in Figure 5. In comparison with the control fruits, the combination treatment extended the shelf life by 8 days, as this figure illustrates. Furthermore, when compared to the control, banana fruit's shelf life might be increased by 3 or 5 days, respectively, by hot water dipping or carrageenan coating alone. In previous findings, Dwivany et al. (2020) reported that carrageenan coating with low‐temperature storage of banana fruit could lengthen the shelf life for 6 days as compared to the control.

The current study found that the combination of hot water treatment and carrageenan coating was effective in extending the shelf life of bananas. According to Suseno et al. (2014), using chitosan coating may help preserve quality and manage decay by delaying the ripening process of Cavendish bananas. Banana fruit's shelf life could thus be extended by a few days. “Gala” apple fruits were stored at 0°C for 8 weeks and 20°C for 7 days as part of their shelf life. Shao et al. (2012) also proposed that a combination of heat treatment at 38°C for 4 days and 1% chitosan coating delayed ripening and lowered decay.

## 4 Conclusion

In this study, the effect of hot water treatment and edible carrageenan coating on banana fruits during cold storage at 13°C and 85%–90% relative humidity was assessed and illustrated. The combination of hot water treatment at 47°C for 5 min and 0.5% carrageenan coating was effective in maintaining physicochemical parameters of banana fruit as well as delaying ripening. Also, it was effective in inhibiting browning of the banana peel surface and decreasing the percentage of disease incidence and severity. Hence, the combination improved the effects of each treatment alone. It was found that the minimum weight loss (%), highest hue value, firmness and titratable acidity (%), lowest total soluble solids (°Brix), and disease incidence were reported to be 10.01%, 87.54 ^o^h, 7.87 N, 0.25%, 18.87 °̊Brix, and 6.50%, respectively, on the 28th day of storage. Moreover, the combined treatment showed the longest shelf life of 36 days, as well as the highest levels of antioxidant activity and total polyphenol content in both the peel and the pulp. Thus, it is possible to effectively use hot water treatment in conjunction with carrageenan coating to enhance the barrier qualities of bananas and increase their shelf life. This study showed that a 0.5% carrageenan coating and a 5‐minute hot water treatment at 47°C increased the shelf life and preserved the quality of bananas while they were being stored in cold storage.

## Author Contributions

**Gemechu Warkina Lencho:** conceptualization (equal), data curation (equal), original draft writing (equal), review and editing (equal). **Nguyen Thi Hanh:** conceptualization (equal), supervision, data curation (equal), original draft writing (equal), review and editing (equal).

## Conflicts of Interest

The authors declare no conflicts of interest.

## Data Availability

Data will be accessible upon request.
